# Whole-Exome Sequencing, Mutational Signature Analysis, and Outcome in Multiple Myeloma—A Pilot Study

**DOI:** 10.3390/ijms252413418

**Published:** 2024-12-14

**Authors:** Lorenz Oelschläger, Axel Künstner, Friederike Frey, Theo Leitner, Lisa Leypoldt, Niklas Reimer, Niklas Gebauer, Lorenz Bastian, Katja Weisel, Verena-Wilbeth Sailer, Christoph Röcken, Wolfram Klapper, Björn Konukiewitz, Eva Maria Murga Penas, Michael Forster, Natalie Schub, Helal M. M. Ahmed, Jutta Kirfel, Nikolas Christian Cornelius von Bubnoff, Hauke Busch, Cyrus Khandanpour

**Affiliations:** 1 Department of Hematology and Oncology, University Medical Center Schleswig-Holstein (UKSH), University Cancer Center Schleswig-Holstein (UCCSH), Campus Lübeck, 23538 Lübeck, Germany; 2 Medical Systems Biology Group, Lübeck Institute of Experimental Dermatology, University of Lübeck, 23538 Lübeck, Germany; 3 University Cancer Center Schleswig-Holstein, University Hospital of Schleswig-Holstein, 23538 Lübeck, Germany; 4 Department of Hematology, Oncology and Bone Marrow Transplantation with Section of Pneumology, University Medical Center Hamburg-Eppendorf, 20521 Hamburg, Germany; 5 Division for Stem Cell Transplantation and Immunotherapy, University Hospital of Schleswig-Holstein, 24105 Kiel, Germany; 6 Department of Pathology, University of Lübeck, 23538 Lübeck, Germany; 7 Department of Pathology, University Medical Center Schleswig-Holstein (UKSH), Campus Kiel, 24105 Kiel, Germany; 8 Institute of Human Genetics, University Hospital Schleswig-Holstein (UKSH)/Christian-Albrechts University Kiel (CAU), 24105 Kiel, Germany; 9 Institute of Clinical Molecular Biology, Christian-Albrechts University, 24105 Kiel, Germany

**Keywords:** multiple myeloma, whole-exome sequencing, somatic signatures

## Abstract

The complex and heterogeneous genomic landscape of multiple myeloma (MM) and many of its clinical and prognostic implications remains to be understood. In other cancers, such as breast cancer, using whole-exome sequencing (WES) and molecular signatures in clinical practice has revolutionized classification, prognostic prediction, and patient management. However, such integration is still in its early stages in MM. In this study, we analyzed WES data from 35 MM patients to identify potential mutational signatures and driver mutations correlated with clinical and cytogenetic characteristics. Our findings confirm the complex mutational spectrum and its impact on previously described ontogenetic and epigenetic pathways. They show TYW1 as a possible new potential driver gene and find no significant associations of mutational signatures with clinical findings. Further studies are needed to strengthen the role of mutational signatures in the clinical context of patients with MM to improve patient management.

## 1. Introduction

Multiple myeloma (MM) is a malignant B-cell neoplasm with an incidence of 1.78 (95% UI 1.69–1.87) per 100,000 and is associated with the Western lifestyle [1,2,3]. MM is often preceded by Monoclonal Gammopathy of Unknown Significance (MGUS) or smoldering multiple myeloma (SMM) and is diagnosed following the International Multiple Myeloma Working Group (IMWG) criteria [2,4,5,6,7]. Treatment is currently guided by patient- and disease-specific factors, such as comorbidities as well as high-risk cytogenetics, which have evolved significantly over the past decade, resulting in improved therapy [8,9].

Recent studies have described the genomic landscape of MM and its minimal residual disease (MRD) and showed genomic differences between progressing and stable myeloma precursor states [10,11,12,13]. With mutational signatures being increasingly analyzed in cancer genomics, the findings may provide insights into cancer biology, prognosis, and even treatment decisions through improved disease classification. Furthermore, a practical guide for analyzing somatic signatures in hematological malignancies has been proposed [14,15,16,17].

While previous studies have shown associations between genomic alterations and patient outcomes in MM [18,19], further studies are needed to investigate the role of somatic signatures in MM. Here, we evaluate the association of somatic signatures with clinical patient characteristics and progression-free survival (PFS) in a cohort of 35 MM patients using whole-exome sequencing (WES). Our results confirm previously reported affected pathways and show new potential driver genes. Significant associations with biological factors or PFS were not found in our study cohort.

## 2. Results

### 2.1. Clinical Characteristics of the Study Group

This study aims to analyze the clinical and genomic features of patients recently diagnosed with MM using clinical and WES data. A total of 35 patients diagnosed with MM between January 2019 and February 2023 were included in this analysis. The median age at diagnosis was 66.8 years, with 42.9% of the patients being male. The majority of patients received bortezomib-based induction therapy, with 17 out of 31 patients (54.8%) subsequently undergoing autologous hematopoietic stem cell transplantation. At the time of analysis, 5 out of the 35 patients (14.3%) had died. Further patient details are shown in Table 1.

### 2.2. Mutational Landscape of Multiple Myeloma Identified by Whole Exome Sequencing

We reconstructed the multiple myeloma mutational landscape from the whole-exome sequencing of the patients’ tumor DNA. Due to the unavailability of matching germline DNA, the identified variants were rigorously filtered, as detailed in the Methods section. In total, 6755 single-nucleotide variants (SNVs) were identified, of which 67.8% were missense mutations and 18.3% were nonsense mutations. Additionally, 13.7% of all SNVs were insertions or deletions, with the remaining variants comprising non-stop and splice site mutations (0.1% each). All samples were microsatellite stable (MSS 0%). In total, 3 out of 35 patients exhibited a high homologous recombination deficiency score (HRD-score > 42), and 12 patients had a high BRCAness score (>20). The average tumor mutational burden (TMB) was 5.05 mutations per megabase (median 3.35; range 1.75–52.20). Genes mutated in more than a third of the cohort included *LILRA5* (54%), *TYW1* (51%), *KMT2C* (40%), *KMT2D* (40%), *NOTCH1* (34%), and *NOTCH2* (34%). All identified variants with PFAM annotations are detailed in Supplementary Table S1.

### 2.3. Potential Driver Genes and Affected Pathways

**Figure 1:**
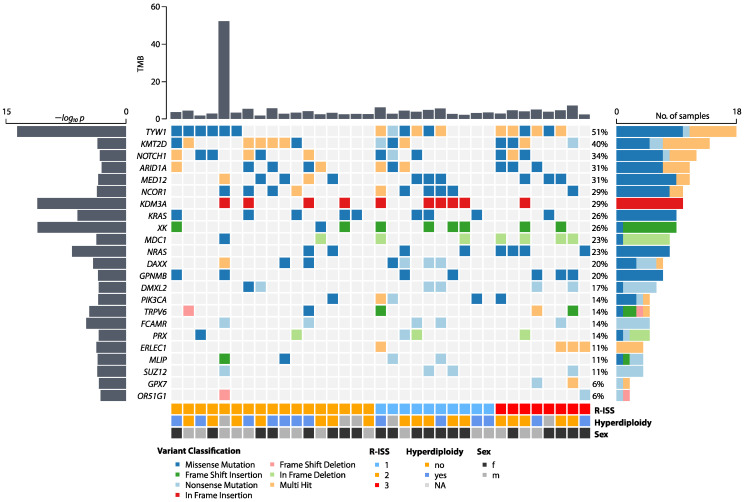
Oncoplot displaying potential driver genes inferred by MutSigCV (*p* < 0.001, *n* = 35). Bar plots refer to individual tumor burden (upper bar plot in mutations per megabase), −*log*_10_ *p* values retrieved from MutSigCV (**left**), and the number of samples harboring mutations in a given gene (**right**). Different classes of mutations are color-coded, and additional covariates are shown below (Revised International Scoring System (R-ISS)).

Identifying potential driver genes in cancer is essential as it offers insights into the underlying molecular mechanisms driving tumorigenesis, informs on potential therapeutic targets, and facilitates the development of personalized treatment strategies to improve patient outcomes. In this study, we employed MutSigCV, a robust computational tool, to identify potential driver genes with high confidence, given its ability to detect significantly mutated genes while minimizing false positives by incorporating patient-specific mutational heterogeneity. Of the 23 potential driver genes (*p* < 0.001), several key driver genes were identified in our cohort, with *TYW1*, *KMT2D*, *NOTCH1*, *ARID1A*, and *MED12* mutations being the most prevalent, found in 31–51% of patient samples. *TYW1* mutations were most frequent, with three variants of missense mutations (*TYW1* c.G393A (*n* = 9), c.R425 (*n* = 15), and c.W437* (*n* = 1)) with allele frequencies between 0.1 and 0.2261. Additionally, our analysis uncovered other mutations previously documented in MM, present in 20–29% of our samples. These include mutations in *NCOR1*, *KDM3A*, *KRAS*, and *NRAS*. We also identified alterations in genes involved in various critical biological processes: *MDC1*, associated with DNA damage response and drug resistance in MM; *DAXX*, involved in chromatin regulation; *GPNMB*, linked to immunosuppression in cancer; and *XK*, which plays a role in hematopoiesis [20,21,22,23,24]. In-frame insertions in *KMD3A* were uniquely observed in our study cohort. *KRAS* and *GPNMB* mutations were solely attributed to missense mutations, whereas *FCAMR* and *SUZ12* mutations were caused exclusively by nonsense mutations. Notably, some of the latter genes have not been described previously in the context of MM, highlighting novel avenues for research. All identified potential driver genes are illustrated in Figure 1, and Supplementary Table S2 shows the complete results from MutSigCV.

**Figure 2:**
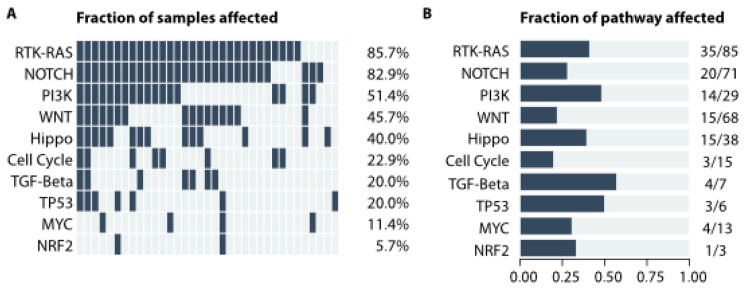
Oncogenic pathways are affected by mutations found in the cohort. (**A**) Heatmap showing the individual sample contributions to affected pathways and the frequency of affected pathways in percentage; (**B**) bar graphs showing the fraction of genes mutated in a particular pathway.

Understanding the oncogenic pathways affected by mutations is crucial for deciphering the mechanisms underlying cancer progression and identifying potential therapeutic targets. With this in mind, we identified pathways influenced by mutations implicated in cancer progression. Figure 2 illustrates known oncogenic pathways affected by mutations, showcasing myeloma-typical pathways pivotal for MM pathogenesis, such as *MAPK* [25,26,27], *NOTCH* [28], *HIPPO* [29], *WNT* [30], *IP3K* [31], *NRF2* [32], *TGF b*, *MYC*, and *TP53* [33]. While some pathways were found to be affected with high frequency, especially *RTK-RAS* and *NOTCH* pathways (see Figure 2A), others were only found in two samples (*NRF2*).

**Figure 3:**
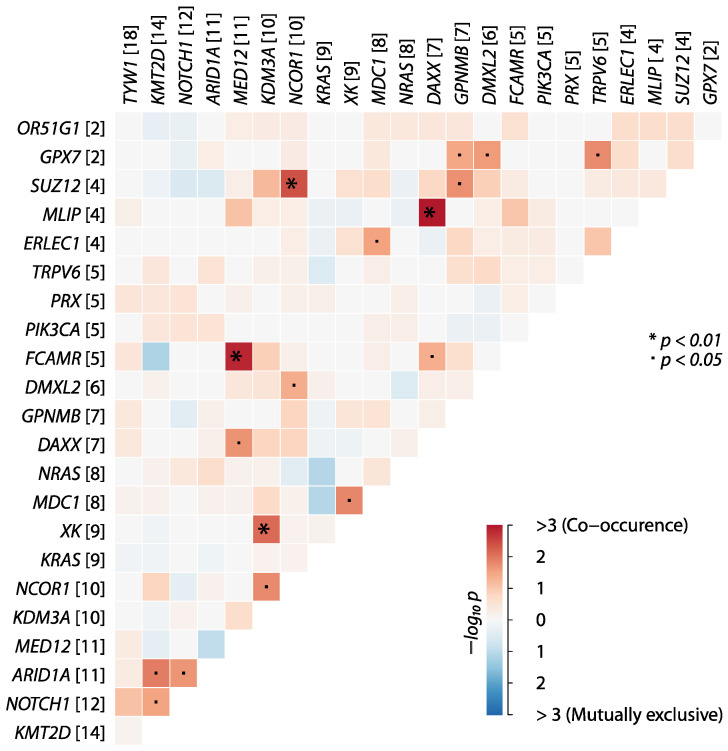
Somatic interactions between mutated genes selected by MutSigCV (*p* < 0.001). Higher co-occurrence of gene mutations is shown in red, while blue refers to mutually exclusive mutations. Gene names on the left and upper side with the number of affected patients in the cohort; *p*-values for statistical significance marked with (*p* < 0.05) or * (*p* < 0.01).

Analyzing somatic interactions is crucial as it unveils the intricate network of genetic interactions driving cancer development and progression, shedding light on potential synergistic or antagonistic relationships between mutated genes. In Figure 3, somatic interactions of genes are displayed. Significant somatic interactions of mutations were found between *FCAMR*/*MED12*, *SUZ12*/*NCOR1*, and *MLIP*/*DAXX* (*p* < 0.01), while others did not frequently co-occur (with blue indicating the exclusivity of mutations and red highlighting the co-occurrence of somatic mutations). The interactions provide further insights into the complex molecular landscape of MM and the interplay between mutated genes.

### 2.4. Analysis of Somatic Signatures

Somatic signatures, which represent distinct patterns of mutations arising from various mutagenic processes, are crucial for understanding the underlying mechanisms of cancer development and progression. Since their first description, they have provided insights into the etiology and chronological evolution of cancers, thus playing an increasingly important role in cancer biology and personalized medicine [34].

**Figure 4:**
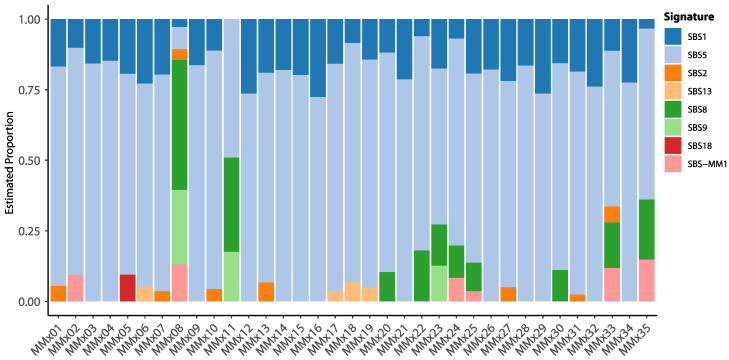
COSMIC single-base substitution (SBS) signatures found in the analyzed cohort. Bar graphs show the color-coded proportion of somatic signatures per individual sample.

All 35 samples were analyzed for their association with somatic signatures using the COSMIC single base substitutions catalog [35] (Figure 4). COSMIC single-base substitutions (SBSs) is a catalog of mutational signatures representing distinct single-nucleotide changes observed in cancer genomes derived from extensive sequencing data across different tumor types. Each SBS signature reflects a unique pattern of mutations linked to specific mutational processes, such as aging, environmental exposures, or DNA repair defects. The most dominant signature identified was single-base substitution 5 (SBS5), with a mean prevalence of 73.2% (s.d. ± 14.3), present in all samples. SBS5, like SBS1, is associated with cell aging (e.g., the clock-like accumulation of mutations) and correlates with patient age. However, our cohort showed no significant correlation between the contribution of SBS5 and the age at diagnosis (linear regression; *p* = 0.2840; multiple R^2^ = 0.0352). This result was corroborated by modeling age as a non-linear trend using b-splines (*p* = 0.3054; multiple R^2^ = 0.0715). Next, we analyzed the impact of somatic signatures on tumor mutational burden (TMB) and homologous recombination deficiency (HRD). TMB was best explained by a linear combination of SBS5, SBS8, SBS9, and SBS-MM1 (stepwise linear regression; *p* = 4 × 10^−4^; adjusted R^2^ = 0.4145), with SBS5, a mutational signature of unknown etiology, having a significant positive correlation (pSBS5 = 0.008). No associations were found for HRD (stepwise linear regression; best model HRD~SBS9; *p* = 0.0817; adjusted R^2^ = 0.0614).

Further, we investigated the associations between mutations in potential driver genes (as depicted in Figure 1) and the estimated somatic signatures. Two genes, *NCOR1* and *GPNMB*, exhibited a significant relationship (*p* < 0.01) between their mutational status and the proportion of somatic signature SBS9 (*NCOR1*; Wilcoxon test; *p* = 0.0054) and SBS8 (*GPNMB*; *p* = 0.0066). SBS9 is associated with non-canonical genome-wide action of activation-induced deaminase (nc-AID). Nc-AID has previously been shown to occur in earlier chronic lymphoid leukemia (CLL) and is associated with IGVH mutational status. Furthermore, *NCOR1* is known to play a role in B-cell development [36,37]. Overall, we identified six genes that showed a significant relationship with the proportions of various somatic signatures (*p* < 0.05), highlighting the complex interplay between specific genetic mutations and mutational patterns. Somatic signatures, association to mutated genes, and biological associations are shown in Table 2. These findings underscore the importance of understanding gene-specific contributions to mutational signatures, which could provide more profound insights into the etiology and progression of MM.

### 2.5. Correlation of Somatic Signatures, Patient Characteristics, Progression-Free Survival (PFS), and Overall Survival (OS)

In our patient samples, no significant associations were found between somatic signatures and age (*p* > 0.05). No significant correlations between cytogenetics (del17p, t(4;14), t(14;16), t(14;20), 1p/1q alteration, t(11;14), and hyperdiploid) at diagnosis and somatic signatures were found (*p* > 0.05). We next investigated the impact of certain mutations, tumor mutational burden, and somatic signatures on progression-free and overall survival. First, we tested for the influence of tumor mutational burden on PFS but found no significant relationship (Univariate Cox regression; beta = 0.014; *p* = 0.22) To investigate the connection between tumor mutational burden and overall survival, a Cox regression model was used, including age at diagnosis, sex, and estimated TMB as independent variables. None of the variables showed a significant correlation with overall survival (*p* > 0.05). However, we observe a trend that higher TMB at diagnosis leads to longer overall survival (Supplementary Figure S2).

**Figure 5:**
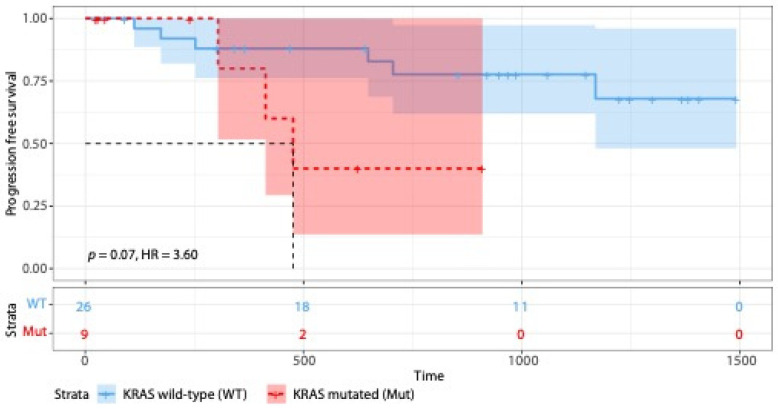
Kaplan–Meier curve differences in progression-free survival with mutational status of *KRAS*.

To assess the clinical impact of somatic signatures, we performed clustering of our patient samples based on clinical data and PFS. Our analysis aimed to determine whether specific somatic signatures were associated with differences in PFS. However, the results indicated that there was no significant correlation between the identified somatic signatures and PFS within our patient cohort. Focusing on the Apolipoprotein B mRNA editing enzyme, catalytic polypeptide (APOBEC) mutational signature (SBS2 and SBS13), a known adverse risk factor in MM, no significant differences in PFS were observed between APOBEC-positive and -negative samples (*p* = 0.47). This suggests that, despite the potential biological relevance of somatic signatures, they may not always directly influence clinical outcomes such as PFS in MM. Our data did not show statistically significant differences in PFS or OS for patients with *TYW1* mutations despite their high occurrence. However, we found that mutations in *KRAS* impact PFS with a median PFS of 304 days in patients with a mutation (*n* = 9) and 940 days in patients not carrying a mutation (*n* = 26; *p* = 0.07; Figure 5); the most frequent mutations in *KRAS* were G13D (*n* = 4), followed by Q61H (*n* = 2) and G12A/R/V (each *n* = 1). Furthermore, we investigated the impact of somatic signature and gene mutations on overall survival. While no impact of somatic signatures was found, mutations in *KRAS*, *NCOR1*, *JAK1*, and *CROCC* were associated with dismal overall survival. The OS analysis, as a separate analysis for patients undergoing autologous stem cell transplantation, is shown in Supplementary Figures S3 and S4.

## 3. Discussion

Our study contributes to the understanding of molecular signatures in MM and adds to the efforts to better understand the implications of genomic alterations in patients with MM. We identified new somatic mutations with high frequency in our cohort (e.g., *TYW1*) with a possible role in pathogenesis and did not find significant correlations of somatic signatures with patient characteristics and clinical outcomes.

In our cohort, 3 of 35 samples (8.57%) showed a high HRD-score, while 12 of 35 (35%) showed a high BRCAness. Previous studies have investigated the role of HRD in MM and the potential use of Poly(ADP-ribose) polymerase (PARP) inhibitors [17,38,39] since PARP enzymes are involved in DNA damage repair. Bortezomib induces a BRCAness state in myeloma cells and impairs the initiation of homologous recombination DNA repair, which may render myeloma cells sensitive to PARP inhibitors [40]. Given the relatively high numbers of HRD and BRCAness, these findings support the potential use of PARP inhibitors.

Intra- and inter-patient genomic heterogeneity has been described in MM patients, as well as variation in potential driver genes [26,41,42]. Our study identified new potential driver genes, including TRNA-YW Synthesizing Protein 1 Homolog (*TYW1*) and Mediator Complex Subunit 12 (*MED12*), and confirmed previously described potential driver genes, e.g., *NRAS*, *KRAS,* and *NOTCH1*.

Interestingly, *TYW1* mutations were present in 18 patients (51%). *TYW1* is a protein-coding gene related to transfer RNA (tRNA) processing, which has not yet been associated with MM. While previous studies have suggested a role of tRNA in tumorigenesis [43,44,45], to our knowledge, our study is the first to report a high frequency of *TYW1* mutations in MM. All mutations are antecedent (c.G393A) or within the radical S-adenosyl methionine (SAM) domain of *TYW1,* which is involved in tRNA modification. *TYW1*—together with six other enzymes (*TYW2-5* and tRNA methyltransferase 5 (*TRMT5*))—catalyze stepwise modifications of tRNA^phe^ at position 37 [46]. While the hypomodification of tRNA^phe^ by the silencing of TYW2 leads to a ribosome frameshift and poorer outcome in colorectal cancer patients [45], the role of *TYW1* mutations on tRNA modification, the resulting translational changes, its implications on tumorigenesis, and its role as a possible therapeutic target in MM need to be studied further.

*MED12* plays an important role in the initiation of transcription and was linked to response in multiple cancers through the regulation of *TGF-β* signaling [47]. *MED12* mutations in the N-terminus were linked to *NOTCH* signaling activation in chronic lymphocytic leukemia, whilst the activation of NOTCH signaling is a promotor of disease progression and forms a supportive microenvironment in MM [48,49], therefore likely to play a role in its pathogenesis. Interestingly, in our analysis, only in-frame insertions were found in *KDM3A*, which acts as an epigenetic regulator via the demethylation of downstream targets contributing to myeloma cell survival [50]. The exact biological effect of these insertions will have to be determined.

Other sub-forms of *KMT2*/*NOTCH*, as well as *ARID1A*, have been previously described in MM or its precursors [11,26,51,52]. Both *KMT2D* and *NOTCH1* mutations are present in various cancer types. *ARID1A*, in most cases, acts as a cancer suppressor, while the loss of *ARID1A* leads to increased cell proliferation [53]. In most samples, the *RTK-RAS*, *NOTCH* pathway, or epigenetic gene regulators were affected by potential driver genes.

The *PI3K/Akt/mTOR* pathway and its impact on MM cell survival and supporting tumor microenvironment have been described [31,54,55]. Particularly, treatment combination with *PIK3CA* inhibitors led to decreased MM cell survival in vitro [56,57]. Other mutations occurring in lower frequencies in our studies, such as *XK*, *TRPV6*, *DAXX*, *DMXL2*, *FCAMR*, and *ERLEC1*, need further investigation to determine their role in MM.

Interestingly, our study revealed a significant interaction between *NCOR1* and *SUZ12* somatic mutations. While *NCOR1* is a transcriptional regulator by bridging repressive transcription factors with chromatin modifiers involved in T-cell survival and B-cell development [37,58], *SUZ12* may be involved in chromatin silencing [59], indicating possible synergistic effects. Further studies must determine the exact mechanisms of somatic interactions and their putative impact on oncogenesis.

SBS5 was present in all patient samples. This finding is well in line with the accumulation of cellular damage, mutations, and changing microenvironment during aging and its possible role in cancer development [60,61,62]. While we found no correlation between age and SBS5 in our study, the distribution of SBS5 may differ while the disease evolves from its precursors. Cytogenetic abnormalities in MM have been linked to therapy response and prognosis [19,63], and the mutational signature of APOBEC is an adverse risk factor in MM [15,16,64,65]. In this study, no association was found between somatic signatures and cytogenetic findings/progression-free survival or overall survival. We found no significant association between TMB and progression-free or overall survival.

Following previous results, [66] we found dismal progression-free survival in patients with KRAS mutations. With 25.7% percent of our study cohort affected by known pathogenic KRAS mutations (G13D, Q61H, and G12A/R/V), our findings underline the possible therapeutic utilities of targeted KRAS inhibitors in patients with MM.

A total of four mutations were associated with dismal overall survival (*KRAS*, *NCOR1*, *JAK1,* and *CROCC*). In contrast to our finding, KRAS expression but not KRAS mutational status was associated with adverse outcomes [67]. The role of the other found mutations on OS in MM is still unclear.

The findings need to be interpreted in consideration of important limitations of our study. Firstly, our sample size is limited. Although, to our knowledge, no differences in methodology in mutation calling were applied, mutation frequencies may be overestimated in our study, considering frequencies in other publications [10,68]. The association between somatic signature, cytogenetics, overall, and progression-free survival might unravel with increasing patient/sample numbers. A recent study developed a new individualized patient outcome prediction model that integrates genomic, clinical, and treatment data [65], but the small sample size in this study does not allow for a similar approach. We focused on the SBS signature as this includes MM-specific signatures (MM1 and MM2). We emphasize that other types of signatures (double-base substitutions (DBSs) and signature of copy number variations (CN)) exist and may provide further insight into the association between somatic signatures and MM. Secondly, due to a lack of material, a correlation with matching germline DNA was not possible. Thirdly, clonal evolution and molecular changes are observed in the majority of patients as the disease progresses and treatment is applied [69,70]. Therefore, somatic signatures change during disease evolution and treatment duration, as previously shown [71]. Our cohort was treated differently, and the median follow-up was relatively short (median 648 days). This limits our findings concerning outcome parameters such as overall and progression-free survival and underscores the need for prospective studies, repetitive analysis, and the correlation of WES data and clinical outcome to evaluate the role of somatic signatures and clinical outcome parameters in MM.

In summary, this study validates the heterogeneous genomic landscape and affected pathways in MM in a real-world clinical setting, identifies new mutations, such as *TYW1*, that have not been previously reported in MM, and describes associations of clinical findings and progression-free survival with somatic signatures. The findings presented in this study have to be confirmed in larger cohorts and may ultimately improve classification and patient management in the future.

## 4. Methods

### 4.1. Case Selection, Extraction of Nucleic Acids, and Whole-Exome Sequencing

Samples from 35 patients with newly diagnosed untreated MM according to the IMWG criteria [1] were collected, and WES analysis was conducted between December 2022 and February 2023. Patients were retrospectively selected for analysis. Clinical data were obtained from patients’ medical records, including radiology reports, oncology/hematology reports, and laboratory data from January 2019 to February 2023. The cut-off date for analysis was 28 February 2023, with a median follow-up of 648 days.

Written informed consent for WES analysis was obtained from all 35 patients. The study was approved by the Institutional Review Board of the University of Lübeck (2024-104) and conducted following the Declaration of Helsinki.

Tumor DNA was extracted from formalin-fixed and paraffin-embedded (FFPE) bone marrow biopsies using the Maxwell FFPE Kit system (Promega, Fitchburg, WI, USA). The quality and quantity of DNA was analyzed using the Qubit system (ThermoFisher, Waltham, MA, USA). Library preparation was carried out using the xGen Exome Hyb Panel v2 (IDT) and the Illumina DNA Prep with Enrichment kit (IDT for Illumina). Sequencing was subsequently performed on the NovaSeq 6000 platform (Illumina, San Diego, CA, USA), with a target sequencing coverage of >400× (tumor).

### 4.2. Sequencing Data Processing, Variant Calling, and Filtering

Raw sequencing data (paired-end fastq files) were mapped to the human reference genome (version GRCh38) and processed using nfcore/sarek (v3.2.3) [72,73]. Briefly, sequencing quality was assessed using fastqc (v0.11.9), and low-quality bases/reads were removed utilizing fastp (v0.23.4) [74]. Next, cleaned reads were mapped to GRCh38 using bwa-mem2 (v2.2.1), and mappings were processed according to GATKs best practices. Variant calling in tumor-only mode was performed using Mutect2 (v4.4.0.0) [75]; identified variants were left-aligned (GATK v4.2.4.1) [76] and variants were annotated using Variant Effect Predictor (VEP v110 [77], GRCh38; adding CADD v1.6 [78], dbNSFP v4.4c, and gnomAD r3.0 as additional annotations) and annotations were converted into *MAF* format using vcf2maf (v1.6.21) [79]; coverage was extracted directly from the INFO field in the vcf files.

Variants outside known coding regions (located in, e.g., intron, UTRs) were removed, and variants with population allele frequency > 0.001 in the GNOMAD or POPFREQ MAX database were discarded, as were variants outside regions defined in the sequencing panel. The top 20 most frequently mutated genes (FLAGS, [80]) were excluded from further analysis to balance artifact removal with retaining true biological signals; the remaining somatic variants were filtered as follows: a minimum coverage of 50, a minimum alternative allele coverage of 5, and a minimum variant allele frequency of 10%. High-impact variants (CADD score > 20) in tumor suppressors and oncogenes, as defined by Vogelstein et al., [81], were filtered such that a minimum coverage of 20, a minimum alternative coverage of 4, and a minimum variant allele frequency of 10% was required (see Supplementary Figure S1). Genes that mutated more often than expected were identified by applying MutSigCV (v1.41 [82]), and potential drivers were identified using a *p*-value threshold of <0.001. Somatic signatures on single-base substitutions (SBSs) were estimated using mmsig [83] (v0.0.0.9000; adjusted for GRCh38) on driver and passenger mutations, and HRD scores were calculated employing the MIRACUM-Pipe (v4.1.0 [84]). Tumor mutational burden (TMB), BRCAness, and microsatellite stability were calculated using the MIRACUM-Pipe (v4.1.0, MSI-Sensor2).

### 4.3. Statistical Analysis

Unless otherwise specified, the analysis and visualizations were conducted using R (version 4.3.2) with the utilization of the following packages: tidyverse (v2.0.0 [85]) for data handling; maftools (v2.17.0, [86]) to summarize, analyze, and visualize variant data; and ComplexHeatmap (v2.16.0) to draw heatmaps.

Progression-free survival (PFS) was calculated from the date of diagnosis and censored at the time of the last clinical contact. PFS analysis, considering potential prognostic factors, was conducted using the Kaplan–Meier method and univariate log-rank test. Furthermore, hazard ratios were determined via a Cox proportional hazards regression model. Overall survival (OS) was calculated from the date of diagnosis using the Kaplan–Meier method and univariate log-rank test for comparison. The survival analysis was executed utilizing the R package’s survivalAnalysis (0.3.0) and survminer (v0.4.9). Survival probability at a certain time was calculated by applying a contsurvplot (v0.2.1) [87]. Associations between mutations in potential driver genes and the estimated somatic signatures were investigated using non-parametric testing (Wilcoxon test). Associations between tumor mutational burden and somatic signatures were calculated using step-wise linear regression.

## Figures and Tables

**Table 1:** Clinical and cytogenetic characteristics of the study cohort.

Sample Size (N)	35
Sex:	
Male *n* (%)	15/35 (42.9)
Female *n* (%)	20/35 (57.1)
Median age at diagnosis [years]	66.8 (Range: 43–85)
Plasma cell bone marrow infiltration (%)	45 (Range: 12–80)
R-ISS-Score:	
R-ISS 1 *n* (%)	10/35 (28.6)
R-ISS 2 *n* (%)	17/35 (48.6)
R-ISS 3 *n* (%)	8/35 (22.9)
Cytogenetics:	
del17p *n* (%)	3/30 (10.0) *
Translocation t(4;14) *n* (%)	0/30 (0) *
Translocation t(14;16) *n* (%)	1/30 (3.3) *
Translocation t(14;20) *n* (%)	1/30 (3.3) *
Initial 1p, 1q alteration *n* (%)	8/30 (26.7) *
Translocation t(11;14) *n* (%)	4/30 (13.3) *
Hyperdiploidy *n* (%)	13/30 (43.3) *
Treatment ** *n* (%)	
VD *n* (%)	6/31 (19.1)
VRD *n* (%)	6/31 (19.4)
VCD *n* (%)	9/31 (29.0)
Dara-VTD *n* (%)	1/31 (3.2)
Dara-VD *n* (%)	1/31 (3.2)
KRD *n* (%)	3/31 (9.7)
RD *n* (%)	3/31 (9.7)
E-KRD *n* (%)	2/31 (6.5)
Autologous transplantation *n* (%)	17/31 (54.8) **
Relapse *n* (%)	5/35 (14.3)
Death *n* (%)	5/35 (14.3)

* 5 Patients with unknown cytogenetic diagnosis; ** 4 Patients with missing clinical data; R-ISS: Revised International Staging System; Dexamethasone (D), Bortezomib (V (Velcade)), Lenalidomide (R (Revlimid)), Cyclophosphamide (C), Daratumumab (Dara), Thalidomide (T), Carfilzomib (K), Elotuzumab (E).

**Table 2:** Genes with significant association with SBS signatures.

Signature	Signature	Gene	*p*-Value
SBS1	Mutations related to cell aging (i.e., clock-like)		
SBS5	Mutations related to cell aging (i.e., clock-like)	*GPNMB*	0.0412
SBS2	Resulting from APOBEC cytidine deaminase activity		
SBS13	Resulting from APOBEC cytidine deaminase activity	*TYW1*	0.0340
SBS8	Unknown etiology	*GPNMP*	0.0066
SBS9	Non-canonical genome-wide action of AID (nc-AID)	*NCOR1* *OR51G1*	0.0054 0.0405
SBS18	Related to DNA damage from reactive oxygen species	*PIK3CA*	0.0179
SBS-MM1	Mutational footprint of melphalan therapy		

## Data Availability

CRAM files have been deposited in the European genome-phenome archive (EGA) under the accession number EGA50000000378. Clinical data are available on request from Cyrus Khandanpour (cyrus.khandanpour@uksh.de).

## Supplementary Materials

The following supporting information can be downloaded at: https://www.mdpi.com/article/10.3390/ijms252413418/s1.

## References

[1] Rajkumar S.V., Dimopoulos M.A., Palumbo A., Blade J., Merlini G., Mateos M.-V., Kumar S., Hillengass J., Kastritis E., Richardson P. (2014). International Myeloma Working Group Updated Criteria for the Diagnosis of Multiple Myeloma. Lancet Oncol..

[2] Rajkumar S.V. (2022). Multiple Myeloma: 2022 Update on Diagnosis, Risk-Stratification and Management. Am. J. Hematol..

[3] Huang J., Chan S.C., Lok V., Zhang L., Lucero-Prisno D.E., Xu W., Zheng Z.-J., Elcarte E., Withers M., Wong M.C.S. (2022). The Epidemiological Landscape of Multiple Myeloma: A Global Cancer Registry Estimate of Disease Burden, Risk Factors, and Temporal Trends. Lancet Haematol..

[4] Landgren O., Kyle R.A., Pfeiffer R.M., Katzmann J.A., Caporaso N.E., Hayes R.B., Dispenzieri A., Kumar S., Clark R.J., Baris D. (2009). Monoclonal Gammopathy of Undetermined Significance (MGUS) Consistently Precedes Multiple Myeloma: A Prospective Study. Blood.

[5] Weiss B.M., Abadie J., Verma P., Howard R.S., Kuehl W.M. (2009). A Monoclonal Gammopathy Precedes Multiple Myeloma in Most Patients. Blood.

[6] Zingone A., Kuehl W.M. (2011). Pathogenesis of Monoclonal Gammopathy of Undetermined Significance and Progression to Multiple Myeloma. Semin. Hematol..

[7] Rajkumar S.V., Landgren O., Mateos M.-V. (2015). Smoldering Multiple Myeloma. Blood.

[8] Palumbo A., Avet-Loiseau H., Oliva S., Lokhorst H.M., Goldschmidt H., Rosinol L., Richardson P., Caltagirone S., Lahuerta J.J., Facon T. (2015). Revised International Staging System for Multiple Myeloma: A Report From International Myeloma Working Group. J. Clin. Oncol..

[9] Branagan A., Lei M., Lou U., Raje N. (2020). Current Treatment Strategies for Multiple Myeloma. JCO Oncol. Pract..

[10] Bolli N., Biancon G., Moarii M., Gimondi S., Li Y., de Philippis C., Maura F., Sathiaseelan V., Tai Y.-T., Mudie L. (2018). Analysis of the Genomic Landscape of Multiple Myeloma Highlights Novel Prognostic Markers and Disease Subgroups. Leukemia.

[11] Oben B., Froyen G., Maclachlan K.H., Leongamornlert D., Abascal F., Zheng-Lin B., Yellapantula V., Derkach A., Geerdens E., Diamond B.T. (2021). Whole-Genome Sequencing Reveals Progressive versus Stable Myeloma Precursor Conditions as Two Distinct Entities. Nat. Commun..

[12] Zátopková M., Ševčíková T., Fanfani V., Chyra Z., Říhová L., Bezděková R., Žihala D., Growková K., Filipová J., Černá L. (2022). Mutation Landscape of Multiple Myeloma Measurable Residual Disease: Identification of Targets for Precision Medicine. Blood Adv..

[13] Manier S., Salem K.Z., Park J., Landau D.A., Getz G., Ghobrial I.M. (2017). Genomic Complexity of Multiple Myeloma and Its Clinical Implications. Nat. Rev. Clin. Oncol..

[14] Davies H., Morganella S., Purdie C.A., Jang S.J., Borgen E., Russnes H., Glodzik D., Zou X., Viari A., Richardson A.L. (2017). Whole-Genome Sequencing Reveals Breast Cancers with Mismatch Repair Deficiency. Cancer Res..

[15] Walker B.A., Wardell C.P., Murison A., Boyle E.M., Begum D.B., Dahir N.M., Proszek P.Z., Melchor L., Pawlyn C., Kaiser M.F. (2015). APOBEC Family Mutational Signatures Are Associated with Poor Prognosis Translocations in Multiple Myeloma. Nat. Commun..

[16] Maura F., Petljak M., Lionetti M., Cifola I., Liang W., Pinatel E., Alexandrov L.B., Fullam A., Martincorena I., Dawson K.J. (2018). Biological and Prognostic Impact of APOBEC-Induced Mutations in the Spectrum of Plasma Cell Dyscrasias and Multiple Myeloma Cell Lines. Leukemia.

[17] Maura F., Degasperi A., Nadeu F., Leongamornlert D., Davies H., Moore L., Royo R., Ziccheddu B., Puente X.S., Avet-Loiseau H. (2019). A Practical Guide for Mutational Signature Analysis in Hematological Malignancies. Nat. Commun..

[18] Samur M.K., Aktas Samur A., Fulciniti M., Szalat R., Han T., Shammas M., Richardson P., Magrangeas F., Minvielle S., Corre J. (2020). Genome-Wide Somatic Alterations in Multiple Myeloma Reveal a Superior Outcome Group. J. Clin. Oncol..

[19] Cardona-Benavides I.J., De Ramón C., Gutiérrez N.C. (2021). Genetic Abnormalities in Multiple Myeloma: Prognostic and Therapeutic Implications. Cells.

[20] Dyer M.A., Qadeer Z.A., Valle-Garcia D., Bernstein E. (2017). ATRX and DAXX: Mechanisms and Mutations. Cold Spring Harb. Perspect. Med..

[21] Sun J., Thingholm T., Højrup P., Rönnstrand L. (2018). XK-Related Protein 5 (XKR5) Is a Novel Negative Regulator of KIT/D816V-Mediated Transformation. Oncogenesis.

[22] Ruff S.E., Logan S.K., Garabedian M.J., Huang T.T. (2020). Roles for MDC1 in Cancer Development and Treatment. DNA Repair.

[23] Lazaratos A.-M., Annis M.G., Siegel P.M. (2022). GPNMB: A Potent Inducer of Immunosuppression in Cancer. Oncogene.

[24] Li X., Wang S., Xie Y., Jiang H., Guo J., Wang Y., Peng Z., Hu M., Wang M., Wang J. (2023). Deacetylation Induced Nuclear Condensation of HP1γ Promotes Multiple Myeloma Drug Resistance. Nat. Commun..

[25] Chapman M.A., Lawrence M.S., Keats J.J., Cibulskis K., Sougnez C., Schinzel A.C., Harview C.L., Brunet J.-P., Ahmann G.J., Adli M. (2011). Initial Genome Sequencing and Analysis of Multiple Myeloma. Nature.

[26] Lohr J.G., Stojanov P., Carter S.L., Cruz-Gordillo P., Lawrence M.S., Auclair D., Sougnez C., Knoechel B., Gould J., Saksena G. (2014). Widespread Genetic Heterogeneity in Multiple Myeloma: Implications for Targeted Therapy. Cancer Cell.

[27] Kortüm K.M., Mai E.K., Hanafiah N.H., Shi C.-X., Zhu Y.-X., Bruins L., Barrio S., Jedlowski P., Merz M., Xu J. (2016). Targeted Sequencing of Refractory Myeloma Reveals a High Incidence of Mutations in CRBN and Ras Pathway Genes. Blood.

[28] Sabol H.M., Delgado-Calle J. (2021). The Multifunctional Role of Notch Signaling in Multiple Myeloma. J. Cancer Metastasis Treat..

[29] Kyriazoglou A., Ntanasis-Stathopoulos I., Terpos E., Fotiou D., Kastritis E., Dimopoulos M.A., Gavriatopoulou M. (2020). Emerging Insights Into the Role of the Hippo Pathway in Multiple Myeloma and Associated Bone Disease. Clin. Lymphoma Myeloma Leuk..

[30] Spaan I., Raymakers R.A., Van De Stolpe A., Peperzak V. (2018). Wnt Signaling in Multiple Myeloma: A Central Player in Disease with Therapeutic Potential. J. Hematol. Oncol..

[31] Ramakrishnan V., Kumar S. (2018). PI3K/AKT/mTOR Pathway in Multiple Myeloma: From Basic Biology to Clinical Promise. Leuk. Lymphoma.

[32] Yen C.-H., Hsu C.-M., Hsiao S.Y., Hsiao H.-H. (2020). Pathogenic Mechanisms of Myeloma Bone Disease and Possible Roles for NRF2. Int. J. Mol. Sci..

[33] Yang P., Qu Y., Wang M., Chu B., Chen W., Zheng Y., Niu T., Qian Z. (2022). Pathogenesis and Treatment of Multiple Myeloma. MedComm.

[34] Alexandrov L.B., Kim J., Haradhvala N.J., Huang M.N., Tian Ng A.W., Wu Y., Boot A., Covington K.R., Gordenin D.A., Bergstrom E.N. (2020). The Repertoire of Mutational Signatures in Human Cancer. Nature.

[35] COSMIC|SBS—Mutational Signatures. https://cancer.sanger.ac.uk/signatures/sbs/.

[36] Kasar S., Kim J., Improgo R., Tiao G., Polak P., Haradhvala N., Lawrence M.S., Kiezun A., Fernandes S.M., Bahl S. (2015). Whole-Genome Sequencing Reveals Activation-Induced Cytidine Deaminase Signatures during Indolent Chronic Lymphocytic Leukaemia Evolution. Nat. Commun..

[37] Lee R.D., Knutson T.P., Munro S.A., Miller J.T., Heltemes-Harris L.M., Mullighan C.G., Jepsen K., Farrar M.A. (2022). Nuclear Corepressors NCOR1/NCOR2 Regulate B Cell Development, Maintain Genomic Integrity and Prevent Transformation. Nat. Immunol..

[38] Pawlyn C., Loehr A., Ashby C., Tytarenko R., Deshpande S., Sun J., Fedorchak K., Mughal T., Davies F.E., Walker B.A. (2018). Loss of Heterozygosity as a Marker of Homologous Repair Deficiency in Multiple Myeloma: A Role for PARP Inhibition?. Leukemia.

[39] Shen H.-Y., Tang H.-L., Zheng Y.-H., Feng J., Dong B.-X., Chen X.-Q. (2022). The PARP1 Inhibitor Niraparib Represses DNA Damage Repair and Synergizes with Temozolomide for Antimyeloma Effects. J. Oncol..

[40] Neri P., Ren L., Gratton K., Stebner E., Johnson J., Klimowicz A., Duggan P., Tassone P., Mansoor A., Stewart D.A. (2011). Bortezomib-Induced “BRCAness” Sensitizes Multiple Myeloma Cells to PARP Inhibitors. Blood.

[41] Rasche L., Chavan S.S., Stephens O.W., Patel P.H., Tytarenko R., Ashby C., Bauer M., Stein C., Deshpande S., Wardell C. (2017). Spatial Genomic Heterogeneity in Multiple Myeloma Revealed by Multi-Region Sequencing. Nat. Commun..

[42] Walker B.A., Mavrommatis K., Wardell C.P., Ashby T.C., Bauer M., Davies F.E., Rosenthal A., Wang H., Qu P., Hoering A. (2018). Identification of Novel Mutational Drivers Reveals Oncogene Dependencies in Multiple Myeloma. Blood.

[43] Huang S., Sun B., Xiong Z., Shu Y., Zhou H., Zhang W., Xiong J., Li Q. (2018). The Dysregulation of tRNAs and tRNA Derivatives in Cancer. J. Exp. Clin. Cancer Res..

[44] Santos M., Fidalgo A., Varanda A.S., Oliveira C., Santos M.A.S. (2019). tRNA Deregulation and Its Consequences in Cancer. Trends Mol. Med..

[45] Rosselló-Tortella M., Llinàs-Arias P., Sakaguchi Y., Miyauchi K., Davalos V., Setien F., Calleja-Cervantes M.E., Piñeyro D., Martínez-Gómez J., Guil S. (2020). Epigenetic Loss of the Transfer RNA-Modifying Enzyme TYW2 Induces Ribosome Frameshifts in Colon Cancer. Proc. Natl. Acad. Sci. USA.

[46] Landgraf B.J., McCarthy E.L., Booker S.J. (2016). Radical *S*-Adenosylmethionine Enzymes in Human Health and Disease. Annu. Rev. Biochem..

[47] Huang S., Hölzel M., Knijnenburg T., Schlicker A., Roepman P., McDermott U., Garnett M., Grernrum W., Sun C., Prahallad A. (2012). MED12 Controls the Response to Multiple Cancer Drugs through Regulation of TGF-β Receptor Signaling. Cell.

[48] Wu B., Słabicki M., Sellner L., Dietrich S., Liu X., Jethwa A., Hüllein J., Walther T., Wagner L., Huang Z. (2017). *MED 12* Mutations and NOTCH Signalling in Chronic Lymphocytic Leukaemia. Br. J. Haematol..

[49] Colombo M., Galletti S., Garavelli S., Platonova N., Paoli A., Basile A., Taiana E., Neri A., Chiaramonte R. (2015). Notch Signaling Deregulation in Multiple Myeloma: A Rational Molecular Target. Oncotarget.

[50] Ohguchi H., Hideshima T., Bhasin M.K., Gorgun G.T., Santo L., Cea M., Samur M.K., Mimura N., Suzuki R., Tai Y.-T. (2016). The KDM3A–KLF2–IRF4 Axis Maintains Myeloma Cell Survival. Nat. Commun..

[51] Bolli N., Avet-Loiseau H., Wedge D.C., Van Loo P., Alexandrov L.B., Martincorena I., Dawson K.J., Iorio F., Nik-Zainal S., Bignell G.R. (2014). Heterogeneity of Genomic Evolution and Mutational Profiles in Multiple Myeloma. Nat. Commun..

[52] Walker B.A., Boyle E.M., Wardell C.P., Murison A., Begum D.B., Dahir N.M., Proszek P.Z., Johnson D.C., Kaiser M.F., Melchor L. (2015). Mutational Spectrum, Copy Number Changes, and Outcome: Results of a Sequencing Study of Patients With Newly Diagnosed Myeloma. J. Clin. Oncol..

[53] Xu S., Tang C. (2021). The Role of ARID1A in Tumors: Tumor Initiation or Tumor Suppression?. Front. Oncol..

[54] Piddock R., Bowles K., Rushworth S. (2017). The Role of PI3K Isoforms in Regulating Bone Marrow Microenvironment Signaling Focusing on Acute Myeloid Leukemia and Multiple Myeloma. Cancers.

[55] García-Ortiz A., Rodríguez-García Y., Encinas J., Maroto-Martín E., Castellano E., Teixidó J., Martínez-López J. (2021). The Role of Tumor Microenvironment in Multiple Myeloma Development and Progression. Cancers.

[56] Hofmann C., Stühmer T., Schmiedl N., Wetzker R., Mottok A., Rosenwald A., Langer C., Zovko J., Chatterjee M., Einsele H. (2014). PI 3K-dependent Multiple Myeloma Cell Survival Is Mediated by the PIK 3 CA Isoform. Br. J. Haematol..

[57] Azab F., Vali S., Abraham J., Potter N., Muz B., De La Puente P., Fiala M., Paasch J., Sultana Z., Tyagi A. (2014). PI3KCA Plays a Major Role in Multiple Myeloma and Its Inhibition with BYL719 Decreases Proliferation, Synergizes with Other Therapies and Overcomes Stroma-Induced Resistance. Br. J. Haematol..

[58] Müller L., Hainberger D., Stolz V., Hamminger P., Hassan H., Preglej T., Boucheron N., Sakaguchi S., Wiegers G.J., Villunger A. (2017). The Corepressor NCOR1 Regulates the Survival of Single-Positive Thymocytes. Sci. Rep..

[59] Rinn J.L., Kertesz M., Wang J.K., Squazzo S.L., Xu X., Brugmann S.A., Goodnough L.H., Helms J.A., Farnham P.J., Segal E. (2007). Functional Demarcation of Active and Silent Chromatin Domains in Human HOX Loci by Noncoding RNAs. Cell.

[60] Berben L., Floris G., Wildiers H., Hatse S. (2021). Cancer and Aging: Two Tightly Interconnected Biological Processes. Cancers.

[61] Urban V.S., Cegledi A., Mikala G. (2023). Multiple Myeloma, a Quintessential Malignant Disease of Aging: A Geroscience Perspective on Pathogenesis and Treatment. GeroScience.

[62] Laconi E., Marongiu F., DeGregori J. (2020). Cancer as a Disease of Old Age: Changing Mutational and Microenvironmental Landscapes. Br. J. Cancer.

[63] Abdallah N., Rajkumar S.V., Greipp P., Kapoor P., Gertz M.A., Dispenzieri A., Baughn L.B., Lacy M.Q., Hayman S.R., Buadi F.K. (2020). Cytogenetic Abnormalities in Multiple Myeloma: Association with Disease Characteristics and Treatment Response. Blood Cancer J..

[64] Grasedieck S., Panahi A., Jarvis M.C., Borzooee F., Harris R.S., Larijani M., Avet-Loiseau H., Samur M., Munshi N., Song K. (2024). Redefining High Risk Multiple Myeloma with an APOBEC/Inflammation-Based Classifier. Leukemia.

[65] Maura F., Rajanna A.R., Ziccheddu B., Poos A.M., Derkach A., Maclachlan K., Durante M., Diamond B., Papadimitriou M., Davies F. (2024). Genomic Classification and Individualized Prognosis in Multiple Myeloma. J. Clin. Oncol..

[66] Chng W.J., Gonzalez-Paz N., Price-Troska T., Jacobus S., Rajkumar S.V., Oken M.M., Kyle R.A., Henderson K.J., Van Wier S., Greipp P. (2008). Clinical and Biological Significance of RAS Mutations in Multiple Myeloma. Leukemia.

[67] Sacco A., Federico C., Todoerti K., Ziccheddu B., Palermo V., Giacomini A., Ravelli C., Maccarinelli F., Bianchi G., Belotti A. (2021). Specific Targeting of the KRAS Mutational Landscape in Myeloma as a Tool to Unveil the Elicited Antitumor Activity. Blood.

[68] Giesen N., Paramasivam N., Toprak U.H., Huebschmann D., Xu J., Uhrig S., Samur M., Bähr S., Fröhlich M., Mughal S.S. (2022). Comprehensive Genomic Analysis of Refractory Multiple Myeloma Reveals a Complex Mutational Landscape Associated with Drug Resistance and Novel Therapeutic Vulnerabilities. Haematologica.

[69] Boyle E.M., Rosenthal A., Wang Y., Farmer P., Rutherford M., Ashby C., Bauer M., Johnson S.K., Wardell C.P., Hoering A. (2021). High-risk Transcriptional Profiles in Multiple Myeloma Are an Acquired Feature That Can Occur in Any Subtype and More Frequently with Each Subsequent Relapse. Br. J. Haematol..

[70] Misund K., Hofste Op Bruinink D., Coward E., Hoogenboezem R.M., Rustad E.H., Sanders M.A., Rye M., Sponaas A.-M., Van Der Holt B., Zweegman S. (2022). Clonal Evolution after Treatment Pressure in Multiple Myeloma: Heterogenous Genomic Aberrations and Transcriptomic Convergence. Leukemia.

[71] Rustad E.H., Yellapantula V., Leongamornlert D., Bolli N., Ledergor G., Nadeu F., Angelopoulos N., Dawson K.J., Mitchell T.J., Osborne R.J. (2020). Timing the Initiation of Multiple Myeloma. Nat. Commun..

[72] Ewels P.A., Peltzer A., Fillinger S., Patel H., Alneberg J., Wilm A., Garcia M.U., Di Tommaso P., Nahnsen S. (2020). The Nf-Core Framework for Community-Curated Bioinformatics Pipelines. Nat. Biotechnol..

[73] Garcia M., Juhos S., Larsson M., Olason P.I., Martin M., Eisfeldt J., DiLorenzo S., Sandgren J., Díaz De Ståhl T., Ewels P. (2020). Sarek: A Portable Workflow for Whole-Genome Sequencing Analysis of Germline and Somatic Variants. F1000Research.

[74] Chen S., Zhou Y., Chen Y., Gu J. (2018). fastp: An Ultra-Fast All-in-One FASTQ Preprocessor. Bioinformatics.

[75] Cibulskis K., Lawrence M.S., Carter S.L., Sivachenko A., Jaffe D., Sougnez C., Gabriel S., Meyerson M., Lander E.S., Getz G. (2013). Sensitive Detection of Somatic Point Mutations in Impure and Heterogeneous Cancer Samples. Nat. Biotechnol..

[76] McKenna A., Hanna M., Banks E., Sivachenko A., Cibulskis K., Kernytsky A., Garimella K., Altshuler D., Gabriel S., Daly M. (2010). The Genome Analysis Toolkit: A MapReduce Framework for Analyzing next-Generation DNA Sequencing Data. Genome Res..

[77] McLaren W., Gil L., Hunt S.E., Riat H.S., Ritchie G.R.S., Thormann A., Flicek P., Cunningham F. (2016). The Ensembl Variant Effect Predictor. Genome Biol..

[78] Rentzsch P., Witten D., Cooper G.M., Shendure J., Kircher M. (2019). CADD: Predicting the Deleteriousness of Variants throughout the Human Genome. Nucleic Acids Res..

[79] Kandoth C., Gao J., Qwangmsk, Mattioni M., Struck A., Boursin Y., Penson A., Chavan S. (2017). Mskcc/Vcf2maf: Vcf2maf v1.6.15. https://zenodo.org/records/1127697.

[80] Shyr C., Tarailo-Graovac M., Gottlieb M., Lee J.J., Van Karnebeek C., Wasserman W.W. (2014). FLAGS, Frequently Mutated Genes in Public Exomes. BMC Med. Genomics.

[81] Vogelstein B., Papadopoulos N., Velculescu V.E., Zhou S., Diaz L.A., Kinzler K.W. (2013). Cancer Genome Landscapes. Science.

[82] Lawrence M.S., Stojanov P., Polak P., Kryukov G.V., Cibulskis K., Sivachenko A., Carter S.L., Stewart C., Mermel C.H., Roberts S.A. (2013). Mutational Heterogeneity in Cancer and the Search for New Cancer-Associated Genes. Nature.

[83] Rustad E.H., Nadeu F., Angelopoulos N., Ziccheddu B., Bolli N., Puente X.S., Campo E., Landgren O., Maura F. (2021). Mmsig: A Fitting Approach to Accurately Identify Somatic Mutational Signatures in Hematological Malignancies. Commun. Biol..

[84] Metzger P., Hess M.E., Blaumeiser A., Pauli T., Schipperges V., Mertes R., Christoph J., Unberath P., Reimer N., Scheible R. (2023). MIRACUM-Pipe: An Adaptable Pipeline for Next-Generation Sequencing Analysis, Reporting, and Visualization for Clinical Decision Making. Cancers.

[85] Wickham H., Averick M., Bryan J., Chang W., McGowan L., François R., Grolemund G., Hayes A., Henry L., Hester J. (2019). Welcome to the Tidyverse. J. Open Source Softw..

[86] Mayakonda A., Lin D.-C., Assenov Y., Plass C., Koeffler H.P. (2018). Maftools: Efficient and Comprehensive Analysis of Somatic Variants in Cancer. Genome Res..

[87] Denz R., Timmesfeld N. (2023). Visualizing the (Causal) Effect of a Continuous Variable on a Time-To-Event Outcome. Epidemiology.

