# Supplementary material for: Whole-Exome Sequencing, Mutational Signature Analysis, and Outcome in Multiple Myeloma—A Pilot Study
Source: Int J Mol Sci. 2024 Dec 14;25(24):13418. doi: 10.3390/ijms252413418 (PMC11680055; doi:10.3390/ijms252413418)
Supplement: Supplementary file 1 [file ijms-25-13418-s001.zip › Supplementary Figure S2. TMB and overall survival.pdf]

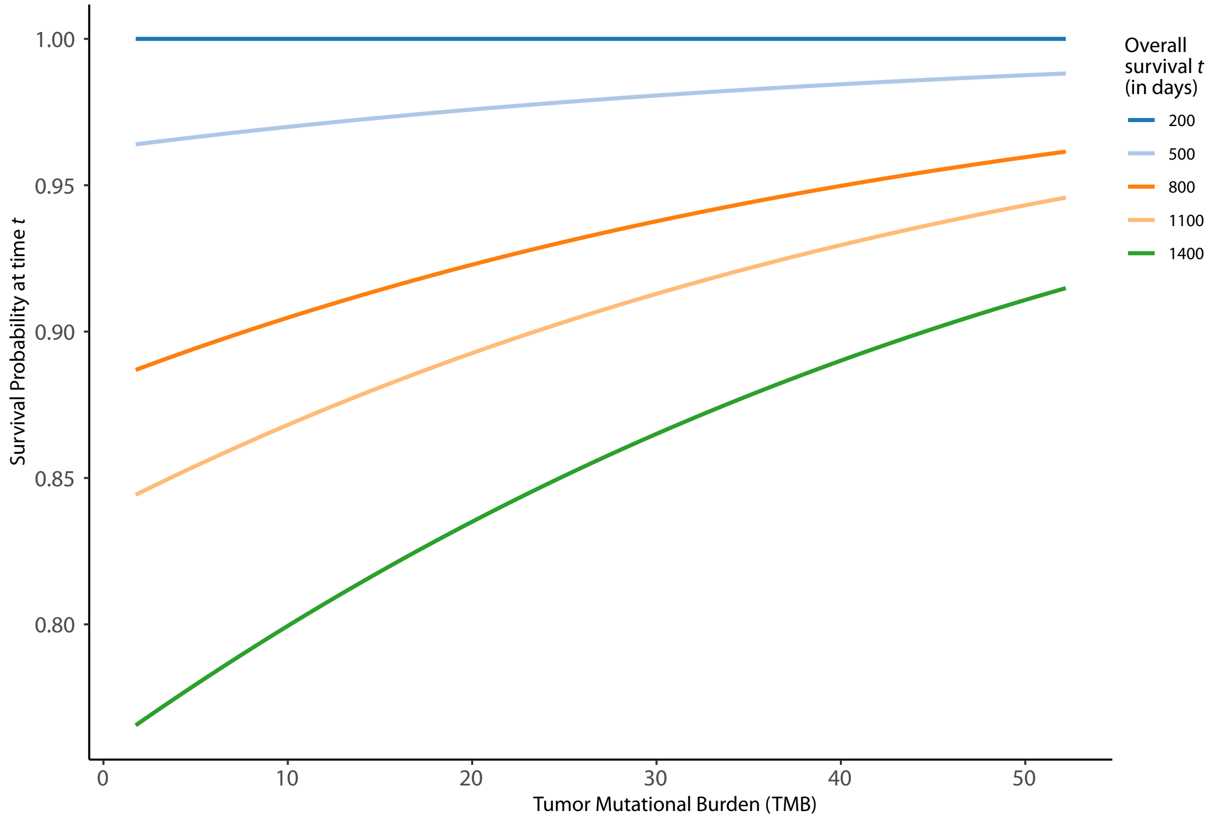

Supplementary Figure S2. Influence of tumor mutational burden (TMB) on survival probability (y-axis) for given time points (200, 500, 800, 1100, 1400 days).
