# Supplementary figures and images for: Whole-Exome Sequencing, Mutational Signature Analysis, and Outcome in Multiple Myeloma—A Pilot Study
Source: Int J Mol Sci. 2024 Dec 14;25(24):13418. doi: 10.3390/ijms252413418 (PMC11680055; doi:10.3390/ijms252413418)

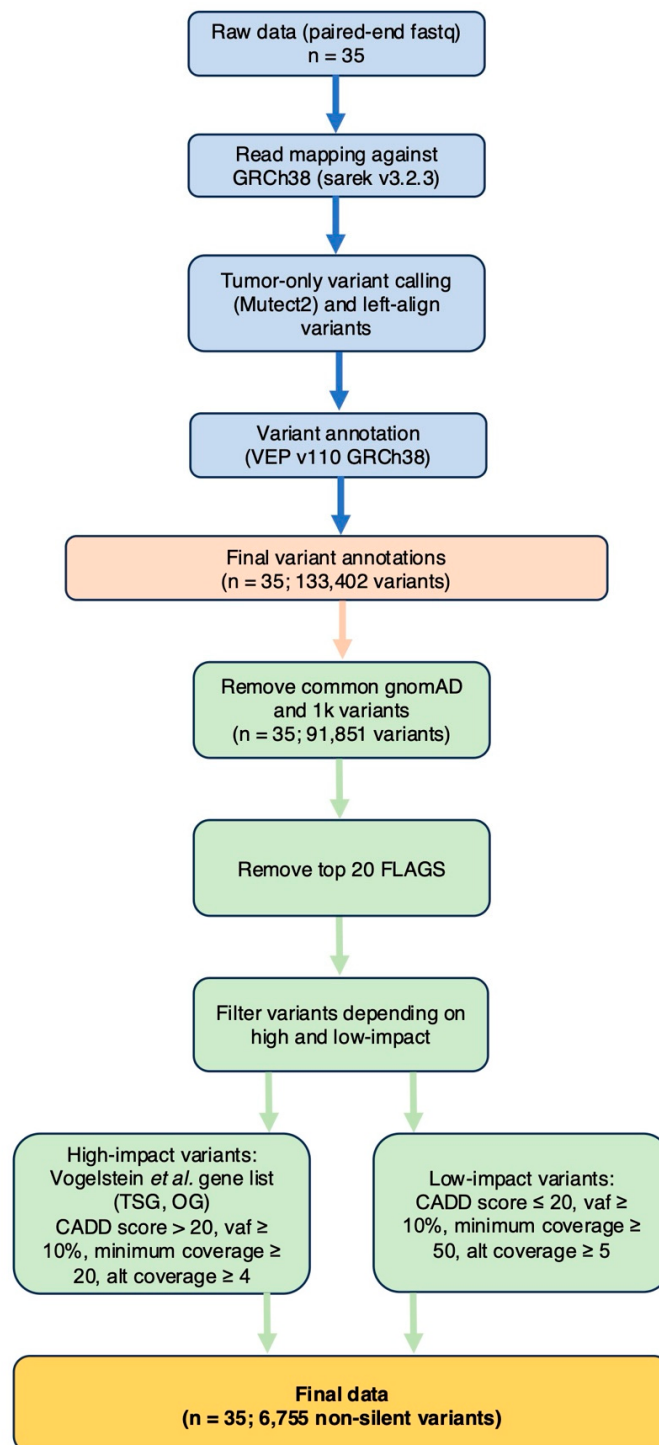

Supplementary Figure S1. Data filtering strategy.

Supplement: Supplementary file 1 [file ijms-25-13418-s001.zip › Supplementary Figure S1.pdf]

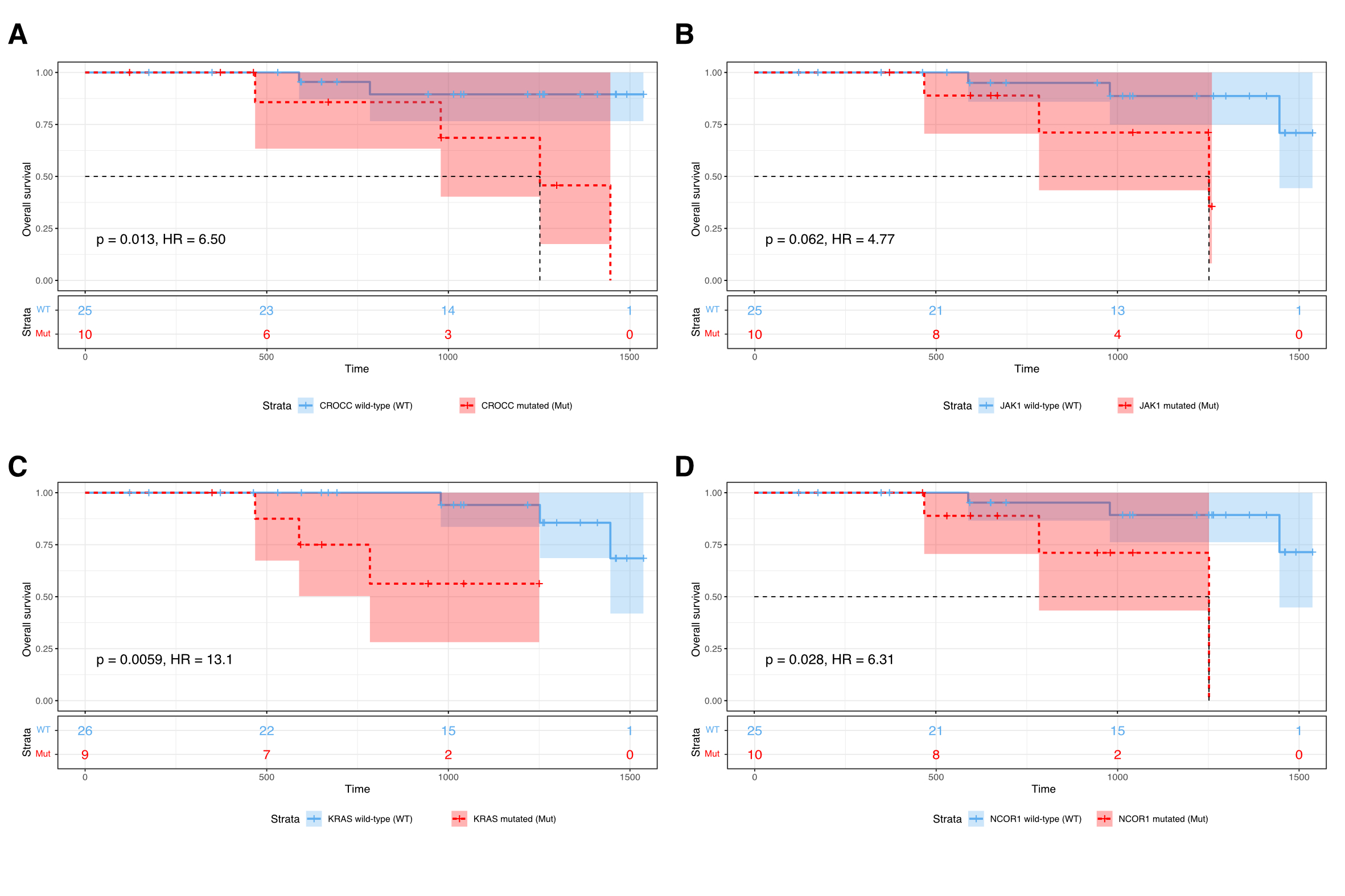

Supplement: Supplementary file 1 [file ijms-25-13418-s001.zip › Supplementary Figure S3. Overall survival (A-D).jpg]

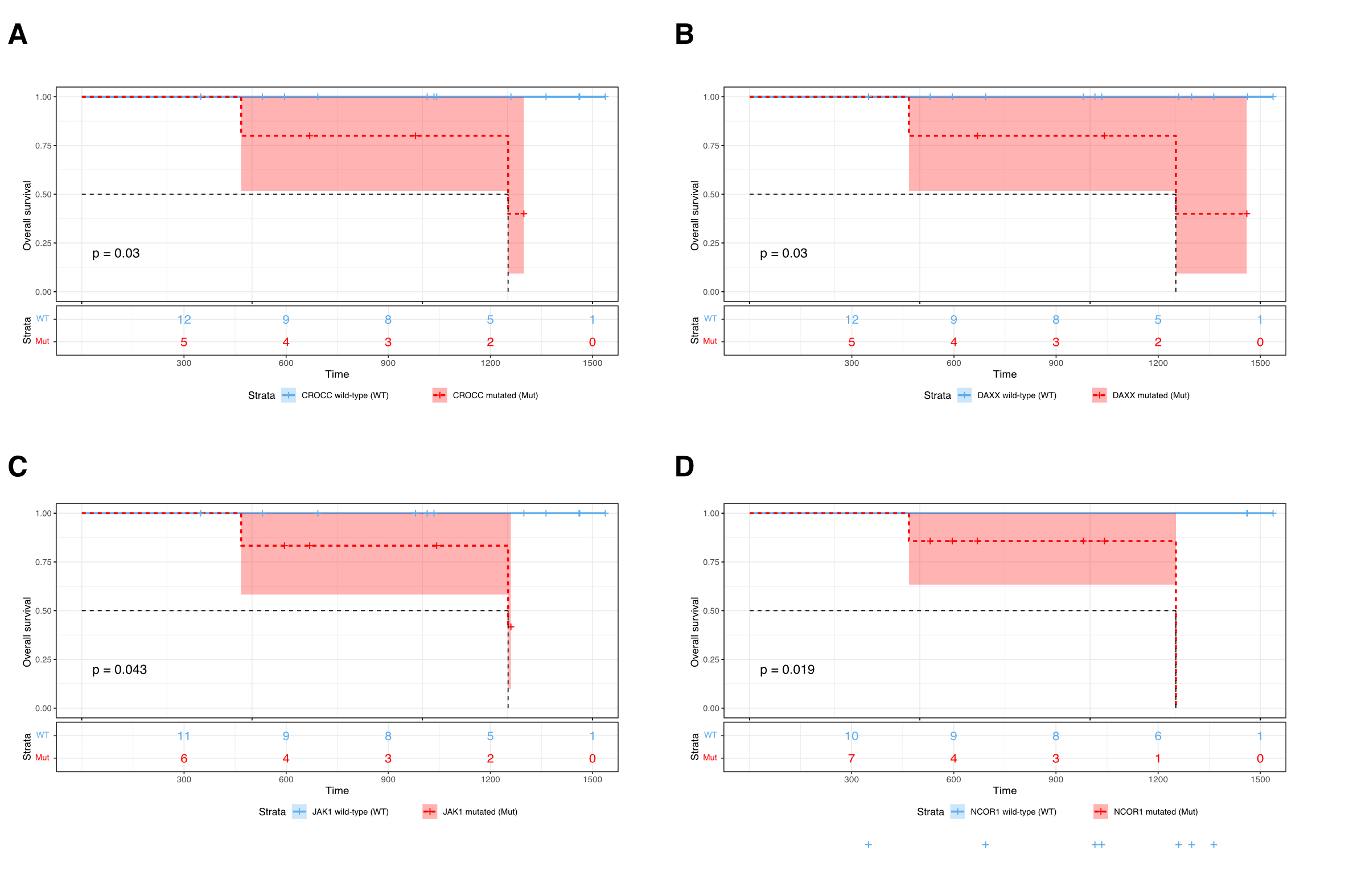

Supplement: Supplementary file 1 [file ijms-25-13418-s001.zip › Supplementary Figure S4. OS Autologous Stem cell Transplantation (A-D).jpg]
